# Unmet information needs and knowledge gaps in cancer patients undergoing oral anticancer therapy

**DOI:** 10.1016/j.rcsop.2025.100678

**Published:** 2025-10-24

**Authors:** Anna K. Moritz, Wolfgang Fehrmann, Markus K. Schuler, Stephanie Stock, Ulrich Jaehde, Nicole Ernstmann

**Affiliations:** a Chair of Health Services Research, Institute of Medical Sociology, Health Services Research, and Rehabilitation Science (IMVR), Faculty of Medicine and University Hospital Cologne, University of Cologne, Cologne, Germany; b Department of Clinical Pharmacy, Institute of Pharmacy, University of Bonn, Bonn, Germany; c Department of Internal Medicine I, University Hospital Carl Gustav Carus, Technical University at Dresden, Dresden, Germany; d Oncology Practice Oskar-Helene Heim, Berlin, Germany; e Institute of Health Economics and Clinical Epidemiology, Faculty of Medicine and University Hospital Cologne, University of Cologne, Cologne, Germany

**Keywords:** Oral anticancer therapy, Information needs, Knowledge gaps, Medication literacy, Information and counseling

## Abstract

**Background:**

The increasing use of oral anticancer therapy (OAT) requires self-management skills from cancer patients. Adequate information and counseling, as well as medication literacy, are key elements of safe and successful therapy in the patient's home.

**Objective:**

The aim of the study was to identify unmet information needs and knowledge gaps of cancer patients regarding their therapy with OAT.

**Methods:**

Semi-structured, guideline-based interviews with cancer patients undergoing OAT were conducted, recorded and professionally transcribed. The transcripts were analysed using qualitative content analysis.

**Results:**

A total of 21 interviews were conducted. Fifteen of the interviewees were female, the median age was 69.6 years. Patients with solid tumours as well as those with blood cancers were interviewed. In the interviews, experiences were reported with various classes of OATs (chemotherapy; anti-hormonal therapy; targeted therapy). The following themes were identified: 1) Therapy-related information needs; 2) No information received; 3) No awareness for information needs; 4) No need for more information; 5) Therapy-related knowledge gaps; 6) Potentially inadequate knowledge. Deficits were identified in relation to correct use, possible interactions, and dealing with adverse events. Individual patients also report actively avoiding information.

**Conclusion:**

The identified information needs and knowledge gaps of patients undergoing OAT highlight the need to optimize information and counseling in order to ensure treatment safety and success. In addition to providing knowledge, the self-management skills of patients should also be specifically strengthened. Furthermore, improvements to the structural framework in the outpatient setting are necessary, particularly with regard to the availability of counseling services. The results can be used as a basis for the development of specific interprofessional educational interventions for those affected.

## Highlights

• Cancer patients needs information for successful and safe therapy with oral anticancer agents at home.
• Affected persons frequently feel insufficiently informed.
• Clear gaps in knowledge about the therapy were identified.
• Results indicate a need for optimization with regard to information and counseling.

## 1 Introduction

There is an increasing use of oral anticancer therapy (OAT) in the last twenty years.^1^ OAT includes all antineoplastic substances that are taken orally, e.g. alkylating agents, anti-hormonal drugs and tyrosine kinase inhibitors. The therapy is conducted in the patient's home environment, which is one of the key differences to intravenous cancer therapy. The outpatient therapy offers numerous advantages for those affected and is the preferred form of therapy for the majority. The administration of medication at home has a lesser impact on the patient's daily life and interpersonal relationships. It allows the cancer patients to gain greater autonomy and an improved quality of life, while reducing doctors' visits and painful injections.^2^, ^3^, ^4^ However, less contact with healthcare professionals and the independent use of medication at home entail challenges for both patients and the healthcare team. Patients take on a high level of self-management skills to ensure therapy success and safety.^5^

They need to know and be able to implement a wide range of specific information regarding their medication. For more than half of the oral anticancer agents, instructions regarding food intake must be observed - as these have an influence on the drug absorption. This can result in a reduction of the therapeutic efficacy or an increase of the risk of toxicity. As with parenteral therapy, OAT can lead to numerous adverse drug reactions. Therefore patients should be informed about possible symptoms so that they can deal with them adequately at home on their own.^1^ In addition, special safety precautions, such as washing hands after handling medication, must be adhered for a number of oral anticancer agents, especially those with cytotoxic effects.^6^, ^7^, ^8^

Given the huge amount of information required, patients need health literacy to correctly and safely self-administer OAT at home. Sörensen's concept of health literacy outlines the essential skills people need to obtain, understand, appraise and apply health-related – especially medication-related - information effectively in order to make informed health decisions.^9^ The concept of medication literacy is a relatively recent evolution of the original concept of health literacy. It is defined as the degree to which individuals can obtain, comprehend, communicate, calculate and process patient-specific information about their medications to make informed medication and health decisions in order to safely and effectively use their medications.^10^ Insufficient health literacy or medication literacy, in particular a lack of knowledge about medication intake, can lead to poor medication-related adherence.^11^^,^^12^ Adherence is defined as the extent to which a person's behaviour, such as taking medication, corresponds with agreed recommendations from a health care provider.^13^ Poor adherence is a common problem, particularly in the field of OAT with adherence rates ranging from 46 % to 100 %.^14^ Low adherence can have a significant negative impact on the success of therapy and patients' quality of life, and also leads to higher healthcare costs.^15^

In Germany, patients usually receive information about the therapy and the prescription of the medication from oncologists in an oncological practice. There, the oncologists should provide patients with comprehensive information about dosage and safety instructions. Some oncological practices also offer a so-called oncological nursing consultation, in which qualified nursing staff provide patients with detailed advice and support during treatment. However, this special consultation is not currently part of standard care and is therefore not refinanced.^16^ Patients usually pick up their medication from the pharmacy themselves. In Germany, patients who are prescribed a new OAT on an outpatient basis are also entitled to an “extended medication consultation” at the pharmacy. The special features of OAT are discussed in a personal consultation. Although this service is refinanced, it is currently only offered by a few pharmacies in Germany. ^17^

Regarding a safe and successful OAT at home, comprehensive education, counseling, and information for patients and, if necessary, their relatives is essential, as emphasized in international guidelines.^18^ To improve patient counseling and information about OAT, it is essential to understand patients' information needs and possible knowledge gaps. However, little is known about these issues. The aim of this study is therefore to explore the information needs and possible knowledge gaps of patients undergoing OAT from the perspective of those affected. However, little is known about these issues, and only few empirical studies have systematically investigated patients' perspectives on information needs and knowledge gaps. The aim of this study is therefore to examine the information needs and possible knowledge gaps of patients undergoing OAT from the perspective of those affected. Given the relational nature of health literacy and medication literacy, information needs and knowledge gaps must be considered in the context of the health care systems. While the present study reflects the German context, the results may manifest differ in other health care systems, particularly in low- and middle-income countries (LMICs) such as India, where structural conditions may pose additional barriers.

## 2 Methods

COREQ guidelines were used to describe the study design, data collection, and data analysis (see Appendix A, Supplementary material 1).^19^

### 2.1 Study design

As part of the AMIKO project (AMIKO = **A**rznei**mi**ttel**ko**mpetenz, German for Medication Literacy), semi-structured interviews were conducted to develop a questionnaire to measure medication literacy in patients undergoing OAT.^1^ In addition to analysing the patients' experience with medication information for developing items in the questionnaire, the information needs and knowledge gaps of the patients were investigated. The present study represents an independent qualitative sub-study, focusing specifically on these issues.

### 2.2 Sample and recruitment

Patients were recruited between June 2023 and January 2024. Recruitment was based on the following inclusion criteria: selected ICD-10 diagnosis (C00–96: Malignant neoplasms, D45: Polycythaemia vera, D46: Myelodysplastic syndromes, D47: Other neoplasms of uncertain or unknown behaviour of lymphoid, haematopoietic and related tissue, D48: Neoplasm of uncertain or unknown behaviour of other and unspecified sites or E24.1: Nelson syndrome), intake of at least one oral anticancer agent, independent use of medication, adult age, and written consent to participate in the study. Exclusion criteria were defined in advance under: off-label therapy, lack of German language skills or other conditions that prevent an interview from being conducted (e.g. dementia), and lack of written consent. Patients were also included when they were classified as survivors but still taking OAT for maintenance. A purposeful sampling strategy was used to include as diverse a group of patients as possible and to reflect different perspectives. Recruitment took place in two oncology practices, each by a study nurse or practice employee who worked there and was trained for the study and who was fully informed about the inclusion criteria. Suitable patients were approached about the study in the waiting area. The contact details of interested patients were then forwarded to the study team. In subsequent telephone calls, the study team used a structured sampling questionnaire to collect specific information on predefined criteria, including age, gender, educational level, diagnosis, OAT class, duration of treatment, and previous experience with OAT. On this basis, attention was paid to achieving the greatest possible variation in the sampling criteria when selecting interview partners in order to reflect a broad spectrum of patient experiences.

### 2.3 Data collection

The interviews were conducted by two researchers from the project team - one male researcher with an approval as pharmacist and one female researcher with a degree in nursing science and health services research. Members of the research group have a wide range of experience in conducting qualitative research, including different methods of data collection and analysis. No prior relationship existed between the interviewer and the participants. The audio-recorded interviews were conducted by phone using a semi-structured guideline. The interview was followed by a short questionnaire with socio-demographic questions on age, gender, highest school-leaving qualification, diagnosis and type of therapy. An excerpt from the interview guideline in Appendix A, Supplementary material 2 shows the main and supporting interview questions on the present research question with regard to possible information needs and knowledge gaps.

### 2.4 Data analysis

The interviews were transcribed and a structured content analysis according to Kuckartz and Rädiker^20^ was conducted using MAXQDA 2022. For the analysis, main categories were deductively formed in the first, initial analysis phases based on the health literacy concept according to Sörensen et al.^9^ Based on their definition, main categories were formed on the topics: 1. receiving information, 2. understanding information, 3. appraising information, 4. applying information, 5. knowledge of OAT and 6. motivation. A second analysis led to the formation of subcategories through an inductive process. In order to identify possible knowledge gaps, the patients' statements were compared with the professional drug-specific information sheets of the DGOP (German Society for Oncological Pharmacy), package inserts of individual drugs and the oncological and pharmaceutical expertise of the project group. The interviews were coded by one researcher. Interviews with an increased need for discussion and in which pharmaceutical expertise was required were counter-coded by a second researcher. The jointly coded interviews were reviewed and discussed by both researchers until a consensus was reached. In cases of disagreement, the researchers jointly reviewed the interview material again and exchanged their individual interpretations until a common understanding was reached. Where necessary, critical passages were also discussed within the project team and with a group of qualitative research colleagues in a research workshop. Passages worthy of discussion particularly concerned statements in which it was not clear whether there was actually a knowledge gap or whether existing knowledge was simply not remembered or recalled during the interview (e.g., when a patient read the name of the medication directly from the packaging—initially coded as a knowledge gap, but later re-evaluated as potentially insufficient knowledge, as the patient demonstrated that they knew where to find the information). These difficulties are described in more detail in the limitations section. The interviews were conducted in parallel with ongoing data analysis. After conducting 21 interviews, no new relevant categories were identified. The data collected showed redundancy in terms of content. It was therefore assumed that theoretical saturation had been reached, so no further interviews were conducted for the research issue.

## 3 Results

### 3.1 Characteristics of the sample

The total amount of the 21 recorded interview material comprises approximately 12 h and 50 min. The average interview length was 37 min (min: 18 min, max: 63 min). On average, the patients were 69.6 years old (range: 28–86). A total of fifteen women and six men were interviewed. Of these, eight had an entrance certificate for a university or university of applied science. In one interview, one relative was also interviewed. Of the interviews, two were employed full time at the time of the interview and twelve of the interviewees lived alone. Both patients with solid tumours (e.g. breast cancer) and blood cancers were interviewed. Each OAT group is represented in the study population. Of the participants, twelve were receiving OAT for the first time. The duration of OAT ranged from less than one year to up to seven years at the time of the interviews. For patients receiving multiple OAT, the total duration was reported, regardless of changes in substances or sequential therapies (Table 1).

**Table 1:** Patient characteristics and therapy-related characteristics (*N* = 21).

**Gender**	
Female	15
Male	6

**Mean age (range)**	69.6 (28–86)
**Living situation**	
Alone	12
With partner / family	9

**Highest qualification**	
University entrance qualification	8
Secondary school certificate	5
Lower secondary school certificate	8

**Job status**	
Not employed: pensioner	16
Not employed: part-time pension	3
Full-time	2

**Diagnosis***(reported by the patient, multiple answers possible)*	
Solid tumours:	
Breast cancer	6
Lung cancer	4
Kidney cancer	2
Colon cancer	1
Ovarian cancer	1
Gastrointestinal stromal tumor (GIST)	1

Blood cancers:	
Myeloproliferative neoplasms (MPNs)	6
Non-Hodgkin lymphoma	2
Myelodysplastic syndrome	1

**Types of therapy***(reported by the patient, multiple answers possible)*	
Chemotherapy (capecitabine, hydroxycarbamide)	7
Anti-hormonal therapy (anastrozole, letrozole, tamoxifen)	6
Targeted therapies (axitinib, crizotinib, dasatinib, imatinib, palbociclib, pazopanib, olaparib, ribociclib, ruxolitinib, sotorasib, venetoclax)	11

**First time undergoing OAT**	
Yes	12
No	9

**Duration of OAT**	
≤ 1 year	9
> 1–5 years	10
> 5 years	2

**Fig. 1:**
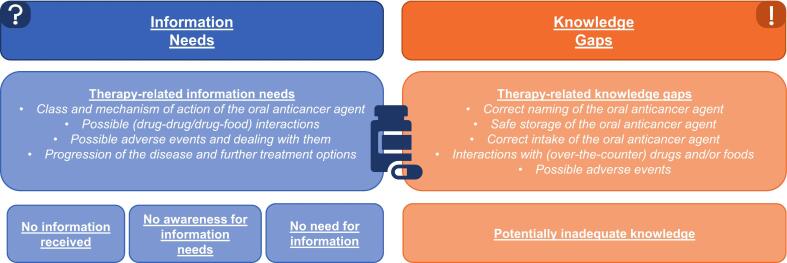
Visualization of the category structure

The two main categories “Information needs” and “Knowledge gaps” with several subcategories are described in the following and illustrated with anchor quotes from the patients interviewed. Text passages in which patients expressed their subjectively perceived information needs regarding OAT were coded under the main category “Information needs.” Statements that indicated incorrect or missing knowledge about OAT were coded under the category “Knowledge gaps.” Passages that could not be clearly assigned to a knowledge gap, but suggested potentially insufficient knowledg**e** regarding therapy, were classified under the category “Potentially inadequate knowledge.” The definitions of the main and subcategories are documented in the code manual (see Appendix A, Supplementary material 3). A visualization of the category structure can be found in Fig. 1.

### 3.2 Main category: Information needs

#### 3.2.1 Therapy-related information needs

In the interviews, patients (18/21) expressed therapy-related information needs and/or the wish for further counseling on various topics:

**Class and mechanism of action of the oral anticancer agent**

Several interviewees (8/21) would like to have more information regarding the effect of the drug on the disease, specifically how exactly the OAT affects the tumor and the entire body. In particular, there is a desire for more explanation on laboratory parameters and the meaning of changes in these values.

*But what now, so to speak/how the mechanism of the drug works, what exactly the drug DOES, […], these details, well, yes, I missed that a bit – right. (51 years, male, OAT: axitinib).*

*Yes, perhaps what specifically affects it. That's what I was missing. I would have liked to know how the drugs affect my tumor. (70 years, male, OAT: sotorasib).*

Possible drug-drug and drug-food interactions

Unmet information needs were also reported by several patients (8/21) with regard to possible interactions of the oral anticancer agents used in the therapy, both with over-the-counter medications and with medications taken daily in the case of co-morbidity. It was sometimes also reported that although there was an awareness of possible interactions, no explicit information was provided in this regard and information had to be researched independently on the internet or in the package leaflet.

*What I don't know sometimes, although I don't take many medications other than these, is whether there would be any reciprocal interactions. No one has ever explained this to me. But I've read a lot about the fact that you have to be really careful. (84 years, female, OAT: letrozole, pazopanib).*

*So I don't get much information from my hematologist, right? He explained to me how my illness, bone marrow, and so on have CHANGED and that I will feel better with this medication, but when it comes to interactions… well, no, actually, no information at all (laughs lightly), I have to say. So the little I know, I just read on the internet and in the package insert. (65 years, female, OAT: ruxolitinib; hydroxicarbamide).*

**Possible adverse events and dealing with them**

Patients also report in various times (9/21), that they were not informed about possible adverse events of the therapy and what to do if symptoms occur. They would have liked more information about this, especially at the beginning of treatment. In this regard, patients reported that instead of being informed about adverse events by the doctor, they were asked to read the patient information leaflet. However, some of the patients also reported that they deliberately avoided reading the package insert because the possible adverse events listed there would frighten them.

*So the doctor didn't tell me about adverse events that COULD occur. I was able to read that myself. […] No, as far as I remember, he didn't do that, he just told me to read through it. (79 years, female, OAT: letrozole).*

*Well, from my hematologist I get pretty much nothing, you know? […] At least not the way I would have wished, let's put it that way, right? That they would really say, ‘This can happen, or that can happen.’ (65 years, female, OAT: ruxolitinib, hydroxycarbamide).*

**Progression of the disease and further treatment options**

Some interviewees (6/21) reported that they had no idea how the therapy would continue and what the chances of success were. They also expressed the wish to be better informed about alternative treatment options, especially in the event that OAT would not be successful.

*The last time I asked “Do I still have a chance at all?”[…] Yes, in GENERAL or like/ I don't know anything. Do I have to take it for the LONG rest of my life […] I don't even know how things will go on with me. (80 years, female, OAT: letrozole).*

*Interviewer (I): …what about side effects? Were you informed about what side effects might occur?*

*Patient (P): (sighs) (…) No, not really. (65 years, female, OAT: ruxolitinib, hydroxycarbamide).*

#### 3.2.2 No information received

A few patients (2/21) expressed general dissatisfaction with the amount of information they received from their physician. According to the interviewees, the information they received during the consultation was often limited to specific instructions on daily intake and no further information was provided.

*I have to be honest with you/ Information is equal to zero. That's only ever when I ask. (80 years, female, OAT: letrozole).*

*Well, actually everything was missing, right? You get prescribed tablets and then the prescription, how often you should take them, and then/ Yes […] Actually EVERYTHING was missing. (65 years, female, OAT: ruxolitinib; hydroxycarbamide).*

#### 3.2.3 No awareness for information needs

Some patients (8/21) were also unable to identify a specific need for information themselves. The lack of awareness of a possible information need is explained by the patients themselves due to their own lay knowledge. In this context, patients also pointed out that they are often unaware of the need for information, especially at the time of the consultation, and that questions only come to mind during the course of treatment. At the beginning, the individual is often overwhelmed and in a state of shock, particularly when the information regarding the therapeutic intervention is provided concurrently with the diagnosis.

*…and anyway, the questions always come to you later, don't they? So if I have questions, then I get an answer, right? It's not like that. But I'm a layperson. So I don't know that much about the disease and, yes, I don't even know what the right questions are to ask, right? (65 years, female, OAT: ruxolitinib; hydroxycarbamide).*

*At that point, I didn't ask any questions. I was so perplexed. (73 years, female, OAT: palbociclib; letrozole).*

#### 3.2.4 No need for more information

Many patients (16/21) reported that they were completely satisfied with the information they had received and that there was no need for further information and counseling

*I would say that what I wanted or NEEDED to know was covered. (79 years, female, OAT: tamoxifen; letrozole).*

*Very good actually […] he explained everything to me in detail. (65 years, male, OAT: hydroxycarbamide).*

However, it should be mentioned that even in interviews where patients expressed satisfaction with the information received, specific information needs and knowledge gaps about the therapy were still identified. Many of the cancer patients (12/21) who felt fully informed also clearly reported that they actively avoided further information - as too much knowledge about the therapy, e.g. with regard to possible adverse events, can also be associated with fears and worries.

*I'm OTHERWISE curious about life, but cancer makes you kind of/ How should I put it? Scared. And the more I know, the more AFRAID I would be. And that's why I often think it's good if I don't know everything. (80 years, female, OAT: letrozole).*

*So I feel better if I don't know the possible side effects/if I don't know them all in the end. (29 years, male, OAT: capecitabine).*

*Do you know what? When I first got cancer, I resolved that I would NOT read anything. I want to be able to continue to live blamelessly and not just drive myself crazy. And that's why I don't read. (80 years, female, OAT: letrozole).*

### 3.3 Main category: Knowledge gaps

In almost all interviews (17/21) with patients undergoing OAT, knowledge gaps and/or potentially inadequate knowledge were identified. The identified knowledge gaps are shown in Table 2. Using anchor quotes, some examples of statements made by the patients are presented and compared with the correct information.

**Table 2:** Knowledge gaps.

Knowledge gap on:	Patient statements	Correct information^a^
Correct naming on the oral anticancer agent (4/21)	*Then I took TermazoFINE […] And NOW I had metastases in my bones again and HE told me to stop taking the Termaz-something. (80 years, female, OAT: letrozole)*	Correct name of the drug is: “Tamoxifen”.

	*The medication is called […] Aliud Pharma. (80 years, female, OAT: letrozole)*	“Aliud Pharma” is a German pharmaceutical company, not the name of the medication.
Safe storage of the oral anticancer agent (8/12)	*So I have a pill box, right? And I always get everything ready for the week […] and when I wake up early in the morning, I first drink a coffee and then I just take them all together (laughs). (65 years, female, OAT: ruxolitinib; hydroxycarbamide)*	OAT should be kept in the original package until the time of intake.

	*No, I wrap them up in paper and then put them in my wallet. (65 years, female, OAT: ruxolitinib; hydroxycarbamide)*	OAT should be kept in the original package until the time of intake.
Correct intake of the oral anticancer agent (8/21)	*These types of capsules are a bit problematic to swallow, […]But by salivating it, you have to keep it in your mouth for a while, then it becomes softer and then I can swallow the tablet well. (80 years, female, OAT: hydroxycarbamide)*	The capsules must not be opened, divided or sucked and must be swallowed whole.

	*So you can't do too much wrong, at least I think so (laughs slightly), I hope. (79 years, female, OAT: venetoclax)*	Various instructions for use to be observed.
Interactions with (over-the-counter) drugs and/or food (12/21)	*I: And what about INTERACTIONS, not SIDE effects, but interactions? Is there anything you need to be aware of, are there any foods or medicines you need to avoid with the tablets?* *P: No. No. I'm not informed of anything. (73* *years, female, OAT: p*albociclib*)*	Existing interactions with grapefruit and St. John's wort.
Possible adverse events (8/21)	*…there are no serious side effects (65 years, male, OAT: hydroxycarbamide)*	Several possible adverse events known.

	*I: And do you know what side effects can occur, what you need to be aware of?* *P: No. (80 years, female, OAT: letrozole)*	Several possible adverse events known.
**Potentially inadequate knowledge** (9/21)	*I: And are there any NUTRIENTS that you may not be allowed to take?* *P: Yes, these are fruits, and indeed/ ask me. (…) Hm (thinking), I can't remember now. The grapefruits, like that, […] cranberries and stuff like that.” (87 years, female, OAT: ribociclib; letrozole)*	Interaction with St. John's wort ➔ Patient may confuse cranberry with currant (similar wording to St. John's wort in German: “Johannisbeere” – “Johanniskraut”).
	*I: …and is there anything you have to do differently with these medications than with other tablets?* *P: No, not really. (73 years, female, OAT: palbociclib; letrozole)*	Various instructions for use: Always take at the same time of day, swallow whole, palbociclib has interactions with grapefruit and St. John's wort, do not repeat a vomited or forgotten dose.

	*I: Is there anything you need to pay particular attention to when dealing with excretion? Have you received any information about the medication?* *P: No, not at all. Not at all. I don't have any problems. (69 years, female, OAT: oplaparid)*	Potential lack of awareness regarding safe handling of excrement: Patients are more likely to associate the question with potential side effects related to the gastrointestinal tract.

a Information based on professional drug-specific information sheets of the DGOP (German Society for Oncological Pharmacy), the package inserts and the oncological and pharmaceutical expertise of the project group.

Various patient information brochures and specialist information contain instructions on the safe handling of the OAT. Patients should wash their hands after handling the medication. Caregivers must wear appropriate protective equipment when handling excreta, vomit or blood. After using the toilet, the toilet lid should be closed and the toilet should be flushed twice.^6^, ^7^, ^8^ Only in one of the interviews analysed here a patient did report washing the hands after handling the medication. A few patients gave answers about handling excrement that were coded as potentially insufficient knowledge. Apart from these cases, no patient mentioned recommended precautions of special handling of excreta or vomit or the safe use of the toilet (closing the lid, flushing twice).

## 4 Discussion and conclusion

### 4.1 Discussion

The aim of the study was to identify unmet information needs based on the reported experiences and descriptions of cancer patients with OAT in qualitative interviews. In addition, the patient statements were compared with specialist information, package inserts and expert statements in order to identify possible knowledge gaps of cancer patients regarding their OAT. Besides, among patients who are completely satisfied with the information they receive, treatment-related information needs and knowledge gaps in various areas were identified.

The present results on the unmet information needs with regard to OAT are consistent with similar international studies in which patients state that they are inadequately or insufficiently informed. The studies by Boons et al.^21^ and Kenis et al.^22^ also revealed a need for information on the medications' effect, potential adverse events and how to manage them, food and drug interactions, and alternative treatment options if the current therapy fails. The reported unmet information needs for OAT are also consistent with general findings on satisfaction with information among cancer patients in connection with other types of therapy. The interviews conducted revealed a wide range of unmet information needs. Additionally, other studies have demonstrated that patients with different types of cancer, undergoing different therapies at different stages of the cancer continuum, have diverse information needs.^23^^,^^24^ Comprehensive knowledge about adverse events is particularly important for those affected.^25^^,^^26^. The relevance of the association between sufficient information on treatment and, in particular, possible adverse events and adherence to medication is clear.^27^, ^28^, ^29^, ^30^, ^31^ Compared to intra venous therapy provided by health professionals in practices and clinics, adherence plays a key role in self-managed OAT at home. Non-adherence in the context of OAT can have significant consequences in terms of treatment success, safety and, therefore, healthcare system costs.^32^, ^33^, ^34^ This highlights the importance of providing comprehensive and personalised information and advice to cancer patients with OAT.

Patients report that they felt insufficiently informed about their OAT. Some of those affected reported that the information and counseling provided was limited to the daily intake instructions. This was also found in a recent scoping review.^35^

Instead of a detailed consultation on possible adverse events or other important topics such as safety precautions, patients also reported that they were only asked to read the package leaflet for the treatment. It should be noted that some patients actively avoid reading the package leaflet, for example to avoid being worried about possible adverse events. Information avoidance in cancer patients is a known phenomenon.^36^^,^^37^ From a psychological perspective, such behaviour can be understood as a coping strategy. For example, avoiding information can be used as a strategy to maintain hope or return to normality by minimizing the perceived impact of the disease and focusing on other aspects of one's life.^36^ This underlines the high relevance of understanding their individual information needs and meeting them in a patient-centred and empathetic manner.

Patients also reported that they were satisfied with the information they received regarding their OAT and that there was no need for further information. However, we were also able to identify knowledge gaps in patients with sufficiently met information needs. A lack of awareness of potentially important information about the therapy becomes clear here.

Comparable results can also be discussed with regard to the identified knowledge gaps. In a cross-sectional survey on patients' knowledge of safe oral anticancer agent handling, explicit knowledge gaps were found. Patients were unaware of important precautions, such as hand washing after handling medication and safe toilet use during therapy.^38^

There were also knowledge gaps, particularly with regard to existing interactions with over-the-counter medications such as St. John's wort, food supplements and food such as grapefruit and grapefruit products. In addition, some of the interviewees had difficulties understanding the question about interactions. There seemed to be a lack of general knowledge and awareness of interactions in medication therapy. Similar to the present study, Arber et al.^39^ also found deficits in knowledge about potential side effects. Knowledge gaps regarding OAT can have far-reaching clinical consequences. A lack of knowledge about drug-drug or drug-food interactions may lead to serious to even lethal adverse events or lead to a weakening of therapeutic efficacy.^1^ With regard to a successful and safe OAT, such knowledge is therefore of central importance and must be fully integrated into the patient education on therapy. Comprehensive knowledge of possible side effects is also required. Patients should be fully aware of the possible side effects in order to be able to react appropriately in terms of risk assessment.^39^ Furthermore, knowledge gaps among patients undergoing OAT may result in unintentional non-adherence, in which medications are not taken or are taken incorrectly due to the lack of knowledge or forgetfulness. This can lead to further adverse events such as preventable toxicities, hospitalizations, or reduced treatment efficacy.^39^, ^40^, ^41^

The identified information needs and knowledge gaps of cancer patients undergoing OAT must be viewed with particular criticality, as supplementary counseling services provided by nursing staff or pharmacies exist in isolated cases but are not widely available and are only partially or not at all refinanced in Germany. According to an analysis by Kaiser et al.^42^ in Germany, in addition to financial burdens, organizational and personnel costs, as well as a lack of engagement or fundamental rejection on the part of physicians, are reasons why nursing consultations in the context of OAT have not yet been adequately implemented. Although “extended medication counseling” in pharmacies is reimbursed, it is only offered by a few pharmacies in Germany. These barriers are related to systemic structures in Germany. At the same time, both national and international intervention studies show that pharmacist- and nurse-led counseling on OAT can be successfully implemented and is associated with improved patient outcomes and safety. Evidence demonstrates that intensive training and support from nursing professionals during OAT can lead to positive outcomes, for example in terms of reducing adverse side effects and treatment interruptions^43^ and promoting adherence rates.^44^ Similarly there is also considerable evidence of positive effects with regard to additional pharmaceutical support during OAT, such as a deeper understanding of side effects,^45^ promotion of adherence,^44^ fewer medication errors, a more positive perception of treatment, and fewer severe side effects.^46^ Additional information and counseling services provided by the multidisciplinary team could specifically address the information needs and knowledge gaps identified in this study, thus improving patient safety and treatment adherence.

The existing body of research suggests that factors such as gender and education level have an impact on medication literacy.^47^ The present study, due to the content analysis method applied, is unable to identify influencing factors. However, the quantitative part of the AMIKO project investigates both factors described in the literature, such as age, gender, and income, as well as less explored factors, such as treatment duration and the use of a medication plan.

### 4.2 Limitations and strengths

This study had several limitations. Only patients from two oncology practices were recruited and interviewed. No patients were interviewed in an inpatient setting like a hospital, where many patients start OAT and receive information. More cancer patients from different practices and facilities should be interviewed about their experiences with information about their OAT. Patients who had only recently started with the OAT were interviewed as well as patients who had been treated undergoing OAT for a longer period of time. This allowed to explore the experiences of patients at different stages of the disease. However, a possible recall bias should therefore be taken into account. Those who had started treatment earlier may not have been able to remember all the information they had received accurately. Therefore, some of the identified knowledge gaps or unmet information needs may be due to memory gaps rather than a lack of information.

Identifying knowledge gaps proved challenging due to the intentionally open design of the interview questions. Closed, test-like questions were avoided to ensure a natural flow of conversation and to avoid uncertainties among the patients. This made it difficult to distinguish between inaccurate statements, behaviour and not mentioning information. For example, only in a few interviews participant explicitly asked about the safe handling of excreta such as stool or vomit, typically when the topic arose naturally during the conversation. Therefore, the absence of statements on this topic cannot be interpreted as a definitive knowledge gap.

In order to increase the validity of the results, the data material was counter-coded and discussed together in an interdisciplinary team. The joint analysis also discussed which information patients need for safe treatment and whether it is sufficient for them to know where to find the relevant details (e.g., the correct medication name or active ingredient). This aspect should be considered when interpreting the results.

The identified information needs and knowledge gaps also do not indicate a potential clinical impact for example on adherence or medication errors. However, studies already show an association between a lack of medication knowledge and adherence to OAT.^31^ Further research is needed here. As part of the quantitative analysis in the AMIKO study, the association between the medication literacy of cancer patients with OAT and adherence will be investigated.

It should also be mentioned that both interviewers have a medical background. This knowledge may have led patients to avoid openly sharing their information needs, uncertainties, and knowledge gaps during the interview. Although efforts were made to ensure a trusting and open atmosphere during the interviews, this potential influence could not be completely excluded and should be taken into account when interpreting the results.

A key strength of the study is the interdisciplinary collaboration with the AMIKO project team. This allowed integration of perspectives from those affected, nursing science, medicine, pharmacy and psychology into results. In addition, independent scientists from various health research disciplines were involved by discussing the results in a methodological workshop.

### 4.3 Conclusion

The present study identifies unmet information needs and knowledge gaps of cancer patients undergoing OAT. Due to reduced contact with healthcare professionals and the need for extensive medication knowledge and self-management, medication literacy is essential for safety and success of treatment. The deficits identified with regard to correct use, potential interactions and management of adverse events highlight the need for targeted training and interventions to systematically improve medication literacy. Individual information needs, including information avoidance, should be taken into account on an individual basis when providing information and counseling. Existing barriers in Germany with regard to the limited availability of multidisciplinary counseling services are currently making it difficult to provide adequate support to patients. The results of this study can serve as a basis for the development and improvement of educational measures for patients undergoing OAT.

### 4.4 Practice implications

To optimize the information and counseling provided to patients undergoing OAT, the relational nature of health and medication literacy must be taken into account. In addition to promoting patients' individual competences and knowledge, attention must be paid to the quality, accessibility, and design of the information provided—tailored to patients' individual needs and supported by an interdisciplinary team (oncologists, nurses, pharmacists, hospitals). Programs such as nursing-led or pharmacist-led counseling services should be developed and implemented across the board. At the systemic level, the refinanced availability of structured counseling services in oncology practices by qualified nursing staff is necessary. The systematic integration of pharmacists into oncological care pathways is also necessary. In addition to providing information, the aim of counseling should also be to promote self-management skills and adherence. At the individual patient level, digital tools (e.g., adherence tools, self-monitoring) can support this. The development and use of easy-to-use patient-facing tools (e.g., digital apps, pictograms, simplified guides, explanatory videos) and the implementation of patient-centered and health literacy-sensitive communication can help achieve these goals and address medication literacy. Previous efforts have placed little emphasis on the concept of medication literacy, which should be integrated more strongly into future activities. The development of counseling services and digital and written support tools should ideally be developed in an interdisciplinary manner and with the participation of those affected in order to capture and include different perspectives and needs.

## CRediT authorship contribution statement

**Anna K. Moritz:** Writing – review & editing, Writing – original draft, Methodology, Investigation, Formal analysis, Data curation, Conceptualization. **Wolfgang Fehrmann:** Writing – review & editing, Methodology, Investigation, Conceptualization. **Markus K. Schuler:** Writing – review & editing, Methodology, Funding acquisition, Conceptualization. **Stephanie Stock:** Writing – review & editing, Supervision. **Ulrich Jaehde:** Writing – review & editing, Methodology, Funding acquisition, Conceptualization. **Nicole Ernstmann:** Writing – review & editing, Supervision, Methodology, Funding acquisition, Conceptualization.

## Ethical approval and consent to participate

Ethical approval for this study was obtained by the Ethics Commission of Cologne University's Faculty of Medicine. All patients were informed verbally and written about the purpose of the study and were asked to sign an informed consent. The privacy rights of the participants were observed.

## Funding

This study is part of the AMIKO project (“Development and validation of a questionnaire to measure medication literacy in patients with oral anticancer therapy”), funded by the 10.13039/501100005972German Cancer Aid (Deutsche Krebshilfe) [grant number 70114882]. Cooperation partners: WINHO (Scientific Institute of Office-Based Hematologists and Oncologists, Cologne, Germany), ILCO e.V. (Self-help organisation for people with a stoma in Germany).

## Declaration of competing interest

The authors declare no conflicts of interest.

## Appendix A Supplementary data

Supplementary material 1 COREQ Checklist Supplementary material 1

Supplementary material 2 Extract from the Interview Guideline. Supplementary material 2

Supplementary material 3 Code Manual - Description of Categories and Subcategories Supplementary material 3

## Footnotes

1 The AMIKO questionnaire, which was developed partly on the basis of the interviews conducted, is currently in the validation phase. The draft questionnaire contains 38 items on medication literacy dimensions such as finding, communicating, understanding or critically analysing. The questionnaire is currently divided into a section for self-assessment of medication literacy and a section for external assessment (performance test). The results of the questionnaire development and validation will be reported in a separate publication.
