# Supplementary material for: Unmet information needs and knowledge gaps in cancer patients undergoing oral anticancer therapy
Source: Explor Res Clin Soc Pharm. 2025 Oct 24;20:100678. doi: 10.1016/j.rcsop.2025.100678 (PMC12712599; doi:10.1016/j.rcsop.2025.100678)
Supplement: Supplementary material 1 — COREQ Checklist [file mmc1.pdf]

Supplementary material 1: COREQ Checklist

Topic	No.	Guide questions	Reported in the following (sub-)chapter:
**Domain 1: Research team and reflexivity**			
Personal characteristics			
Interviewer/facilitator	1	Which author/s conducted the interview or focus group?	▪ Methods (Data collection), Declarations (Author contribution)
Credentials	2	What were the researcher's credentials? E.g., PhD, MD	▪ Methods (Data collection)
Occupation	3	What was their occupation at the time of the study?	▪ Methods (Data collection), Declarations (Author contribution)
Gender	4	Was the researcher male or female?	▪ Methods (Data collection)
Experience and training	5	What experience or training did the researcher have?	▪ Methods (Data collection), Declarations (Author contribution)
Relationship with participants			
Relationship established	6	Was a relationship established prior to study commencement?	▪ Methods (Data collection)
Participant knowledge of the interviewer	7	What did the participants know about the researcher? E.g., personal goals, reasons for doing the research	▪ Ethical approval and consent to participate
Interviewer characteristics	8	What characteristics were reported about the interviewer/facilitator? E.g., Bias, assumptions, reasons, and interests in the research topic	▪ Methods (Data collection)
**Domain 2: Study design**			
Theoretical framework			
Methodological orientation and theory	9	What methodological orientation was stated to underpin the study? E.g., grounded theory, discourse analysis, ethnography, phenomenology, content analysis	▪ Methods (Data analysis)
Participant selection			
Sampling	10	How were participants selected? E.g., purposive, convenience, consecutive, snowball	▪ Methods (Sample and Recruitment)
Method of approach	11	How were participants approached? E.g., face-to-face, telephone, mail, email	▪ Methods (Sample and Recruitment; Data collection)
Sample size	12	How many participants were in the study?	▪ Results (Characteristics of the sample)
Non-participation	13	How many people refused to participate or dropped out? Reasons?	▪ N/A
Setting			
Setting of data collection	14	Where was the data collected? E.g., home, clinic, workplace	▪ Declarations (Author contribution)
Presence of non-participants	15	Was anyone else present besides the participants and researchers?	▪ Declarations (Author contribution)
Description of sample	16	What are the important characteristics of the sample? E.g., demographic data, date	▪ Results (Characteristics of the sample)
Data collection			
Interview guide	17	Were questions, prompts, guides provided by the authors? Was it pilot tested?	▪ Methods (Data collection)

Repeat interviews	18	Were repeat interviews carried out? If yes, how many?	▪ N/A
Audio/visual recording	19	Did the research use audio or visual recording to collect the data?	▪ Methods (Data collection)
Field notes	20	Were field notes made during and/or after the interview or focus group?	▪ Declarations (Author contribution)
Duration	21	What was the duration of the interviews or focus group?	▪ Results (Characteristics of the sample)
Data saturation	22	Was data saturation discussed?	▪ Methods (Data analysis), Results (Characteristics of the sample)
Transcripts returned	23	Were transcripts returned to participants for comment and/or correction?	▪ N/A
**Domain 3: Analysis and findings**			
Data analysis			
Number of data coders	24	How many data coders coded the data?	▪ Methods (Data analysis), Declarations (Author contribution)
Description of the coding tree	25	Did authors provide a description of the coding tree?	▪ Table 3 Overview of the category system
Derivation of themes	26	Were themes identified in advance or derived from the data?	▪ Methods (Data analysis)
Software	27	What software, if applicable, was used to manage the data?	▪ Methods (Data analysis)
Participant checking	28	Did participants provide feedback on the findings?	▪ N/A
Reporting			
Quotations presented	29	Were participant quotations presented to illustrate the themes/findings? Was each quotation identified? E.g., participant number	▪ Results (Main category Information needs, all sub-chapters)<br>▪ Results (Main category Knowledge gaps, Table 3)
Data findings consistent	30	Was there consistency between the data presented and the findings?	▪ Results (All sub-chapters)<br>▪ Discussion and conclusion (Discussion)
Clarity of major themes	31	Were major themes clearly presented in the findings?	▪ Results (All sub-chapters)<br>▪ Discussion and conclusion (Discussion)
Clarity of minor themes	32	Is there a description of diverse cases or discussion of minor themes?	▪ Results (All sub-chapters)<br>▪ Discussion and conclusion (Discussion)

Adaption from: **Tong A, Sainsbury P, Craig J. Consolidated criteria for reporting qualitative research (COREQ): a 32-item checklist for interviews and focus groups. *Int J Qual Health Care*. 2007 Sep 16;19(6):349–57.**
