# Supplementary material for: Unmet information needs and knowledge gaps in cancer patients undergoing oral anticancer therapy
Source: Explor Res Clin Soc Pharm. 2025 Oct 24;20:100678. doi: 10.1016/j.rcsop.2025.100678 (PMC12712599; doi:10.1016/j.rcsop.2025.100678)
Supplement: Supplementary material 2 — Extract from the Interview Guideline. [file mmc2.pdf]

Supplementary material 2: Extract from the interview guideline

Main questions	Supporting questions
**1. Initial question / medication intake**<br>Could you start by briefly telling me the name of the medication you are taking?<br>Can you please tell me exactly how you take your medication?	- What do you do differently from other medications? / What do you pay attention to? *For example: Medication instructions; Storage/disposal; Dealing with excretions; Handling in case of forgetting/vomiting*<br>- How do you handle the medication when you are not at home?
**2. Drug-drug/Drug-food interactions**<br>To what extent do you pay attention to interactions with your cancer medication?	- Are there any foods you need to avoid?<br>- Are there any medications you need to avoid?
**3. Medication prescription**<br>Can you tell me how the conversation went when the doctor prescribed your cancer medication?	- To what extent were you informed of other treatment options?<br>- To what extent were you informed about possible adverse events?
**4. Information needs**<br>If you remember the first day you came home with your cancer medication...did you feel well informed and educated?<br>[Alternatively: Did you know the things you need to know when taking your medication?]	- Were you able to apply the information you received in your everyday life?<br>- What information did you miss?<br>- How would you have liked to receive information and advice about your medication?<br>- Are you missing further persons to contact on specific questions?
