# Supplementary material for: Unmet information needs and knowledge gaps in cancer patients undergoing oral anticancer therapy
Source: Explor Res Clin Soc Pharm. 2025 Oct 24;20:100678. doi: 10.1016/j.rcsop.2025.100678 (PMC12712599; doi:10.1016/j.rcsop.2025.100678)
Supplement: Supplementary material 3 — Code Manual - Description of Categories and Subcategories [file mmc3.docx]

**Supplementary material 3: Code Manual - Description of Categories and Subcategories**

| **Main**  **categories** | **Subcategories and topics** | **Definition** |
| --- | --- | --- |
| **Information needs** | Therapy-related information needs | This category includes statements in which patients express their subjective perceived information needs regarding OAT. Text passages are coded if patients state that they did not understand the information (sufficiently), would have liked more information, or would have found more information helpful.  Distinction: Statements that are incorrect in terms of content, without an explicitly expressed desire for information, are not recorded here but are recorded under the main category knowledge gap. |
|  | - *Class and mechanism of action of the oral anticancer agent* | This category includes statements from patients that reveal a need for information regarding the mode of action and/or pharmacological class of OAT. This includes, for example, the desire for a detailed explanation of how the drug works in the body, what function it performs, what connections exist between laboratory values and medication intake, as well as a basic understanding that it is a cancer drug. |
|  | - *Possible (drug-drug/drug-food) interactions* | This category includes statements made by patients that reveal a need for information about possible interactions (with other medications and foods) with the OAT they are taking. This also includes expressed uncertainties that require independent research if this information was not provided during the consultation with the physician. |
|  | - *Possible adverse events and dealing with them* | This category includes statements from patients expressing a need for information about possible adverse effects of the OAT they are taking. This also includes the desire foradviceson how to deal with symptoms when they occur. |
|  | - *Progression of the disease and further treatment options* | This category includes statements from patients expressing a need for information about possible adverse effects of the OAT they are taking. This also includes the desire for recommendations on how to deal with symptoms when they occur. |
|  | No information received | This category includes statements in which patients report general dissatisfaction and feel inadequately informed or not informed at all about OAT. |
|  | No awareness for information needs | This category includes statements in which patients are not aware of their own need for information, or are only partially aware of it, or are unable to perceive it due to overwhelm, shock, or lack of prior knowledge - as a result of which patients do not know what questions to ask or what information to request. |
|  | No need for more information | This category includes statements in which patients express that they feel sufficiently informed, do not require further information, and/or consciously refrain from receiving further information, e.g., due to fear of worrying information (e.g., possible side effects). |
| **Knowledge gaps** | Therapy-related knowledge gaps | This category includes statements from patients that indicate incorrect or missing knowledge about OAT. In contrast to the subjectively perceived information need, this refers to objectively identified knowledge gaps—when key aspects of the therapy (dosage instructions, side effects, or interactions) are unknown or misunderstood. The knowledge gaps can be explicitly mentioned by patients or implicitly identified from outside, through incorrect statements or perceived uncertainties in conversation. |
|  | - *Correct naming of the oral anticancer agent* | This category includes statements indicating that patients do not know the name of the OAT correctly, clear uncertainties in pronunciation are also coded. |
|  | - *Safe storage of the oral anticancer agent* | This category includes statements that indicate a lack of knowledge about the proper storage of OAT. This includes, for example, inappropriate storage (too humid/too warm, etc.) and/or a lack of knowledge about safe storage in everyday life/in the household (e.g., the use of collecting boxes). |
|  | - *Correct intake of the oral anticancer agent* | This category includes statements that reveal gaps in knowledge about the correct use of OAT, such as when to take it, whether to take it with food, and how to swallow the medication correctly. |
|  | - *Interactions with (over-the-counter) drugs and/or foods* | This category includes statements that reveal a lack of knowledge about possible interactions between OAT and other medications or foods. |
|  | - *Possible adverse events* | This category includes statements that reveal a lack of knowledge about adverse effects or in which patients underestimate or misjudge the potential risk. It also includes a lack of knowledge about how to deal with adverse effects when they occur. |
|  | Potentially inadequate knowledge | This category includes statements in which no clear knowledge gap can be identified, but there are indications of potentially insufficient knowledge. |
